# mRNA-Based Nanomedicinal Products to Address Corneal Inflammation by Interleukin-10 Supplementation

**DOI:** 10.3390/pharmaceutics13091472

**Published:** 2021-09-15

**Authors:** Itziar Gómez-Aguado, Julen Rodríguez-Castejón, Marina Beraza-Millor, Mónica Vicente-Pascual, Alicia Rodríguez-Gascón, Sara Garelli, Luigi Battaglia, Ana del Pozo-Rodríguez, María Ángeles Solinís

**Affiliations:** 1 Pharmacokinetic, Nanotechnology and Gene Therapy Group (PharmaNanoGene), Faculty of Pharmacy, Centro de Investigación Lascaray Ikergunea, University of the Basque Country UPV/EHU, Paseo de la Universidad 7, 01006 Vitoria-Gasteiz, Spain; itziar.gomez@ehu.eus (I.G.-A.); julen.rodriguez@ehu.eus (J.R.-C.); marina.beraza@ehu.eus (M.B.-M.); monica.vicente@ehu.eus (M.V.-P.); alicia.rodriguez@ehu.eus (A.R.-G.); 2 Bioaraba, Microbiology, Infectious Disease, Antimicrobial Agents, and Gene Therapy, 01006 Vitoria-Gasteiz, Spain; 3 Dipartimento di Scienza e Tecnologia del Farmaco, Università degli Studi di Torino, Via Pietro Giuria 9, 10125 Torino, Italy; saragarelli.sg@gmail.com (S.G.); luigi.battaglia@unito.it (L.B.)

**Keywords:** messenger RNA, solid lipid nanoparticles, interleukin-10, corneal inflammation, polyvinyl alcohol, topical administration, gene augmentation, transfection, corneal epithelium, advanced therapies

## Abstract

The anti-inflammatory cytokine Interleukin-10 (IL-10) is considered an efficient treatment for corneal inflammation, in spite of its short half-life and poor eye bioavailability. In the present work, mRNA-based nanomedicinal products based on solid lipid nanoparticles (SLNs) were developed in order to produce IL-10 to treat corneal inflammation. mRNA encoding green fluorescent protein (GFP) or human IL-10 was complexed with different SLNs and ligands. After, physicochemical characterization, transfection efficacy, intracellular disposition, cellular uptake and IL-10 expression of the nanosystems were evaluated *in vitro* in human corneal epithelial (HCE-2) cells. Energy-dependent mechanisms favoured HCE-2 transfection, whereas protein production was influenced by energy-independent uptake mechanisms. Nanovectors with a mean particle size between 94 and 348 nm and a positive superficial charge were formulated as eye drops containing 1% (*w*/*v*) of polyvinyl alcohol (PVA) with 7.1–7.5 pH. After three days of topical administration to mice, all formulations produced GFP in the corneal epithelium of mice. SLNs allowed the obtaining of a higher transfection efficiency than naked mRNA. All formulations produce IL-10, and the interleukin was even observed in the deeper layers of the epithelium of mice depending on the formulation. This work shows the potential application of mRNA-SLN-based nanosystems to address corneal inflammation by gene augmentation therapy.

## 1. Introduction

The eye is considered the perfect target for gene therapy due to its physiological features: it is easy to access and examine, it has a well-defined anatomy and it is relatively immune privileged. Moreover, from an experimental point of view, in the same subject, one eye can be used as a control, whereas the other eye can be used as the experimental target [1,2,3]. Currently, 47 clinical trials restricted to “ocular diseases” are registered in the database of Gene Therapy Clinical Trials Worldwide [4], all of them targeting retinal degenerative diseases, except one, which concerns corneal opacity. The cornea is a transparent tissue localized in the anterior segment of the eye, which contributes to eyesight by focusing a visual image through light refraction. Several factors can damage this tissue and provoke corneal inflammation or keratitis, such as infections, dry eye, eyelid disorders, physical and chemical damage, and a wide variety of underlying diseases [5]. Current treatments of corneal inflammation are based on corticosteroids, which require repeated topical applications and have been associated with multiple adverse effects, such as infectious keratitis, increased intraocular pressure, and cataracts [6]. Alternatively, the topical administration of IL-10, an anti-inflammatory cytokine, has been suggested as an effective treatment for corneal inflammation [7]. However, the short half-life and poor eye bioavailability of IL-10 limit its therapeutic use. Therefore, IL-10 ocular delivery may be better attempted by gene therapy, with the aim of inducing IL-10 de novo synthesis in corneal cells [8].

In the last few decades, a wide range of reagents and techniques suitable to transfer nucleic acids into cells have been developed, in order to modulate both *in vitro* and *in vivo* gene expression. Particularly, mRNA therapeutics present several advantages: since mRNA does not need to enter inside the cell nucleus in order to exert its effect, protein translation is faster than in the case of DNA; furthermore, mRNA expression is temporary and it owns a safe profile with no risk of insertion-related mutagenesis, as it does not incorporate into the genome of the host [9]. Additionally, the production and manufacturing of mRNA is easy to scale up in a cost-effective, standardized, and reproducible way. However, mRNA instability is one of its major vulnerabilities [10], which makes the development of suitable delivery systems necessary. Delivery systems should be able to protect mRNA against RNase degradation, and to ensure its intracellular delivery inside the cytoplasm of the target cell [11]. In this context, different strategies and efforts for advances in nanotechnology and material sciences are still ongoing [9]. Presently, non-viral vectors are at the frontline of mRNA therapy, in contrast to DNA-based gene therapy; particularly, lipid-based vectors are among the most extensively used non-viral nucleic acid delivery platforms. Indeed, mRNA therapy has gained much attention after its application in the vaccination against the SARS-CoV-2 [12,13,14]. It should be noted that during the COVID-19 pandemic, the first vaccines approved for distribution and use against the SARS-CoV-2 by the European Medicines Agency (EMA) and the United States Food and Drug Administration (FDA) were based on mRNA encapsulated in lipid nanoparticles (LNs) [15,16]. These mRNA vaccines have been well-tolerated and have demonstrated approximately 95% efficacy against COVID-19, with few adverse events [17,18].

Among lipid-based systems, liposomes and solid lipid nanoparticles (SLNs) have been suggested as up-and-coming non-viral ocular drug delivery systems. [19,20]. Liposomes were introduced as carriers for the delivery of nucleic acids for gene therapy over two decades ago and, to date, they still represent the most widely studied vectors for gene delivery. Liposomes used for gene delivery are typically nanometric and are defined by a spherical vesicle with an aqueous internal cavity enclosed by a lipid bilayer membrane. However, these systems are not devoid of stability and efficacy issues [21,22]. SLNs were more recently developed with the aim of, among other things, addressing the above issues underlying liposome gene transfection [23]. SLNs are spherical particles with a solid lipid core matrix, which is stabilized by surfactants in an aqueous dispersion, and are usually composed of well-tolerated physiological lipids [24,25,26,27]. In addition, SLNs have demonstrated to be effective for ocular topical drug administration [28]. This administration route shows several advantages: it is non-invasive, drug absorption into the systemic circulation is minimized, first pass metabolism is prevented, and formulations are easy to administer. However, the main issue of conventional ocular drug delivery systems relies on the poor retention onto the ocular surface, due to corneal clearance; as a consequence, the amount of drug that is able to cross the cornea and access the ocular structures is small. Therefore, an effective ocular drug delivery system should increase corneal retention time and improve drug permeation through the cornea [29]. SLNs possess a nanometer sized-range, lipophilic properties, and usually positive surface charges. This makes them suitable for topical drug administration by improving corneal permeation and retention [30,31]. Previously, our research group has used a gene augmentation strategy to induce IL-10 production into the cornea by the administration of SLNs containing plasmid DNA (pDNA) [32]. However, mRNA could be an alternative therapeutic option to tackle corneal inflammation, thanks to its high efficacy, safety profile, and versatility for fast protein production.

The aim of this experimental work is the development of SLNs containing mRNA as non-viral vectors, to be used as a genetic supplementation strategy for the *de novo* production of IL-10 in corneal cells. Additionally, vectors prepared with plasmid DNA (pDNA) were evaluated for comparison purposes. Formulations containing mRNA or pDNA encoding green fluorescent protein (GFP) or IL-10 were characterized from a physico-chemical point of view, and then evaluated for internalization and transfection properties, both *in vitro* by using human corneal epithelial (HCE-2), and *in vivo* after administration to mice by ocular instillation.

## 2. Materials and Methods

### 2.1. Materials

1,2-Dioleoyl-3-trimethylammonium-propane chloride salt (DOTAP) was obtained from Avanti Polar-lipids, Inc. (Alabaster, AL, USA). Precirol^®^ ATO 5 (glyceryl palmitostearate) was kindly provided by Gattefossé (Madrid, Spain). Tween 80 and dichloromethane were purchased from Panreac (Madrid, Spain) and sodium behenate from Nu-Chek Prep (Eleysian, AL, USA). Sigma-Aldrich (Madrid, Spain) provided protamine sulfate salt from salmon (Grade X) (P), dextran (Mn of 3260 Da) (DX), DEAE-dextran, Nile Red and partially hydrolyzed polyvinyl alcohol (PVA) 9000–10,000 Da M_*w*_. Lifecore Biomedical (Chaska, MN, USA) supplied hyaluronic acid (HA) (M_*w*_ of 100 KDa).

CleanCap^TM^ EGFP mRNA (5moU) and CleanCap^TM^ Cyanine 5 EGFP mRNA (5moU were acquired from TriLink BioTechnologies (San Diego, CA, USA). Both of the nucleic acids encode green fluorescent protein (GFP). mRNA encoding human IL-10 was customized by TriLink BioTechnologies. Professor BHF Weber laboratory (University of Regensburg, Germany) kindly provided plasmid pcDNA3-EGFP (6.1 kb) encoding GFP. Plasmid pUNO1-hIL10 (3.7 kb), which encodes human IL-10, was acquired by InvivoGen (San Diego, CA, USA). Label IT^®^ Cy^®^5 Nucleic Acid Labeling Kit was obtained from Mirus Bio (Madison, WI, USA).

Materials used on agarose gel in an electrophoresis assay were acquired from Bio-Rad (Madrid, Spain). Ambion^TM^ RNase I was purchased from Life Technologies (Thermo Fisher Scientific, Madrid, Spain), Deoxyribonuclease I (DNase I) and sodium dodecyl sulfate (SDS) from Sigma-Aldrich and GelRed^TM^ from Biotium (Fremont, CA, USA).

Human Corneal Epithelial (HCE-2) cells were purchased from American Type Culture Collection (ATCC, Manassas, VA, USA). Cell culture reagents, including Dulbecco’s Modified Eagle´s Medium/Nutrient Mixture F-12 with GlutaMAX^TM^ (DMEM/F-12 with GlutaMAX^TM^), fetal bovine serum (FBS), Penicillin-Streptomycin, Trypsin/EDTA and Attachment Factor were acquired from Life Technologies (Thermo Fisher Scientific, Madrid, Spain), whereas epidermal Growth Factor (EGF) and human insulin solution were obtained from Myltenyi Biotec (Madrid, Spain) and Sigma-Aldrich (Madrid, Spain), respectively. The DuoSet Ancillary reagent kit and Enzyme-linked immunoassay (ELISA) for IL-10 were obtained from R&D Systems (Minneapolis, MN, USA).

Reporter lysis buffer was provided by Promega Biotech Ibérica (Madrid, Spain). Paraformaldehyde (PFA) was obtained from Panreac, while 40,6-diamidine-20-phenylindole dihydrochloride (DAPI)-fluoromount-G was purchased from Southern Biotech (Birmingham, AL, USA). Phosphate buffered saline (PBS) and HEPES buffered solution were obtained from Gibco (Thermo Fisher Scientific, Madrid, Spain) and Lipofectamine^TM^ 2000 Lipid-Reagent was acquired from Life Technologies (Thermo Fisher Scientific, Madrid, Spain). The 7-Amino-Actinomycin D (7-AAD) Viability Dye was acquired from Beckman Coulter (Brea, CA, USA).

Antibodies used for immunoassay, Anti-GFP polyclonal antibody and goat anti-Rabbit IgG (H+L) Cross-Adsorbed Secondary Antibody Alexa Fluor 488, were provided by Life Technologies (Thermo Fisher Scientific, Madrid, Spain), except rabbit Anti-IL-10 antibody, which was obtained from Abcam (Cambridge, UK). Sakura Finetek Europe (Alphen aan den Rijn, The Netherlands) supplied Tissue-Tek^®^ O.C.T.-compound.

Other chemicals, unless detailed, were reagent grade from Panreac (Barcelona, Spain) and Sigma-Aldrich (Madrid, Spain).

### 2.2. Formulation of SLNs and Vectors

Three different techniques were used in order to prepare SLNs: solvent evaporation/emulsification (SLN_EE_), hot-melt emulsification (SLN_HM_) and coacervation (SLN_C_).

SLN_EE_ were elaborated as previously reported [33], with the solid lipid Precirol^®^ ATO 5 and the cationic lipid DOTAP, stabilized by the surfactant Tween 80. Briefly, SLN_EE_ were obtained by the sonication (Branson Sonifier 250, Danbury) of the organic phase (Precirol^®^ ATO 5 dissolved in dichloromethane 5% *w*/*v*) in the aqueous phase, containing the cationic lipid DOTAP (0.4% *w*/*v*) and Tween 80 (0.1% *w*/*v*). SLNs’ precipitation occurred upon dichloromethane evaporation.

SLN_HM_ was produced as a SLN_EE_ with minor modifications. Briefly, DOTAP (0.4% *w*/*v*) and Tween 80 (0.1% *w*/*v*) were dissolved in water. This aqueous solution and Precirol^®^ ATO 5 were heated in a bain-marie. When both phases were led to 80 °C and lipids melted, the aqueous solution was added to the Precirol^®^ ATO 5. The emulsion was obtained by sonication (Branson Sonifier 250, Danbury, CT, USA) for 30 min at 50 W. SLNs were obtained by cooling the obtained nanoemulsion on ice for 30 min. 

SLN_C_ were composed of behenic acid as the lipid matrix, coated by the suspending agent PVA 9000 and the cationizing agent DEAE-dextran, as previously described [32]. Briefly, sodium behenate and PVA 9000 were dissolved in water under hot agitation, and when the solution reached 80 °C and became translucent, NaOH was added. Then, when the solution turned completely transparent DEAE-dextran was added drop by drop, turning the mixture turbid. Later, HCl was rapidly added and when the suspension turned white, it was cooled under stirring in a water bath. The final product underwent a re-melting process, by heating again and cooling by agitation in a water bath.

When required, Nile Red was incorporated in the preparation of all SLNs to label them. In the case of SLN_EE_, Nile Red was dissolved in the dichloromethane of the organic phase, whereas in SLN_HM_ Nile Red was dissolved in dichloromethane and added during the first 30 s of the sonication step. Finally, in the case of SLN_C_, a solution of dichloromethane containing Nile Red was added during the re-melting step.

In order to prepare SLN-based vectors, an aqueous solution of protamine (P) was added to the nucleic acid, mRNA or pDNA. Then, a solution of polysaccharide, dextran (DX) or hyaluronic acid (HA) dissolved in water was added. Finally, the SLN suspension was added to the previous complexes and incubated for 20 min. Electrostatic interactions between negative and positive charges of the components led to the formation of the final vectors, in which complexes were adsorbed on the surface of the cationic SLNs.

The weight ratios of the components of the formulations are summarized in Table 1.

In order to obtain viscous formulations suitable for ocular delivery to animal models, an aqueous solution of PVA (85,000–124,000 M_*w*_) was incorporated into the above mentioned vectors. The final concentration of PVA was 1% (*w*/*v*).

### 2.3. Characterization of SLNs and Vectors: Size, Polydispersity Index and ζ-Potential Measurements

The mean size and polydispersity index (PDI) of 3 batches of SLNs and vectors were measured by Dynamic Light Scattering (DLS) and ζ-potential was determined by Laser Doppler Velocimetry (LDP). These measurements were carried out in a ZetaSizer Nano ZS (Malvern Panalytical, Malvern, UK) after the appropriate dilution of the samples in Milli-Q™ water (EDM Millipore, MA, USA).

### 2.4. Agarose Gel Electrophoresis Assay

Studies of mRNA binding capacity, protection from RNase I digestion and release from the vectors were performed in a 1.2% agarose gel electrophoresis labelled with Gel Red^TM^. The gel ran for 60 min at 75 V, then it was immediately analyzed with the Uvitec Uvidoc D-55-LCD-20 M Auto transilluminator (Cambridge, UK). In order to evaluate binding capacity, vectors were diluted in MilliQ^TM^ water to a final concentration of 0.12 µg mRNA/µL in the gel. Protection from RNase I digestion was analyzed by the addition of 6 U RNase I/µg mRNA; mixtures were incubated at 37 °C for 40 min in a heater. Then, the samples were removed from the heater and mixed with an SDS solution (final concentration of 1%), at room temperature. The same SDS solution was added to the vectors to unbind the mRNA in the release studies. Two controls for the integrity of the mRNA were included in the assay: RiboRuler High Range RNA Ladder and untreated CleanCap^TM^ EGFP mRNA (5moU).

In order to study the pDNA binding capacity, protection against DNase I digestion and release from the vectors, 0.7% agarose gel electrophoresis with Gel Red^TM^ was used. The gel was analyzed with the Uvitec Uvidoc D-55-LCD-20 M Auto transilluminator (Cambridge, UK) after running for 30 min at 120 V. The binding capacity was evaluated by adding vectors diluted in MilliQ^TM^ water to a final concentration of 0.03 µg pDNA/µL in the gel. For DNAse I protection, the same concentration was exposed to 1 U DNase I/2.5 μg pDNA and then incubated at 37 °C for 30 min in a heater. The samples were removed from the heater and mixed with an SDS solution (4%) to a final concentration of 1% at room temperature. The same SDS solution was added to the vectors to unbind the plasmid in the release studies. Two controls for the integrity of the pDNA were included in the gels: 1 kb pDNA ladder from NIPPON Genetics Europe (Dueren, Germany) and untreated pcDNA3-EGFP plasmid.

### 2.5. pH Measurement

Crison Basic 20 pH meter (Crison Instruments, Barcelona, Spain) was employed to determine the pH of the vectors. Measures were carried out in triplicate and the pH meter was calibrated daily.

### 2.6. Cell Culture Studies

Human corneal epithelial (HCE-2) cell line was used in order to perform *in vitro* studies. HCE-2 cell line was maintained in a DMEM/F-12 GlutaMAX^TM^ medium, which was supplemented with 15% (*v*/*v*) heat-inactivated fetal bovine serum (FBS), insulin (4 mg/mL), Epidermal Growth Factor (EGF) (10 ng/mL), and Penicillin–Streptomycin (1% *v*/*v*) and incubated at 37 °C with 5% CO_2_. Cells were sub-cultured once a week using Trypsin/EDTA in flasks earlier treated with Attachment Factor. The medium was renewed every 2 days. Cells from passages 1 to 4 were used to perform all *in vitro* assays.

#### 2.6.1. Transfection Efficacy and Cell Viability

mRNA-based vectors were prepared 72 h before their addition and maintained at 4 °C before their use.

After incubation with Attachment Factor, the cells were cultured on 24-well plates for 72 h at a density of 70,000 cells/well, until the cells were adhered and were able to create a monolayer. Later, part of the medium was removed, leaving enough volume to cover the cells, and a total volume of 75 µL of each vector diluted in Hank’s Balanced Salt solution (HBS) (equivalent to 2.5 µg) was added to each well for 4 h in the incubator at 37 °C in a 5% CO_2_. The medium containing the vectors was removed after the incubation time, and the cells were refreshed with 1 mL of the complete medium. The cells were kept growing during 48 h or 72 h in the case of mRNA- or pDNA-vectors, respectively.

The percentage of transfected cells and intensity of fluorescence, indicative of the amount of GFP produced, as well as cell viability, were measured using a CytoFLEX flow cytometer (Beckman Coulter). For this purpose, the cells were washed with 500 µL of PBS and then detached by incubation with 300 µL of Trypsin/EDTA for 5 min. After the centrifugation of cell suspension at 1000 rpm for 5 min, the supernatant was removed, and the pellet of cells was resuspended in 500 µL of PBS. Ten thousand events were collected for each sample. Transfection efficacy was measured at 525 nm (FITC) [34], and cell viability was determined at 610 nm (ECD), after the addition of 7-Amino-Actinomycin D (7-AAD) Viability Dye to the samples. The percentage of transfected cells was calculated counting the positive fluorescent GFP cells over the total cells. The intensity of fluorescence represented the mean of the intensity of fluorescence per labelled cell, which is correlated with gene expression and protein production [35,36,37].

The effect of temperature on cell transfection was studied by the incubation of HCE-2 cells at 4 °C for 30 min, prior to the addition of the vectors. The cells were maintained for 4 h at 4 °C, and then the cells were treated as explained above.

#### 2.6.2. Cellular Uptake

The internalization of the vectors by the HCE-2 cells was studied by using vectors containing SLNs labelled with the fluorescent Nile Red dye (λ = 590 nm), as previously described [8] and as explained above. The vectors were prepared 72 h before their addition. For this purpose, a total volume of 75 µL of each vector diluted in HBS (equivalent to 2.5 µg of nucleic acid) was added to each well; then, the cells were incubated for 2 h at 37 °C in a 5% CO_2_. Once the incubation time had finished, the culture medium was removed and the cells were detached from the plates, as described in Section 2.6.1, for the cytometry analysis of transfected cells. The entrance of the vectors was analyzed by using a CytoFLEX flow cytometer (Beckman Coulter) at 610 nm (ECD). For each sample, 10,000 events were collected.

Additionally, the effect of temperature on cellular uptake was studied by the incubation of cells at 4 °C for 30 min before the incorporation of the vectors. Once the Nile Red-labelled vectors were added to the cell cultures, the cells were maintained at 4 °C for an additional 2 h. Finally, the cells were collected to evaluate the vector uptake by flow cytometry, as described above. In this case, the percentage of positive cells corresponds to the cells that have uptaken vectors labelled with Nile Red over the total cells.

#### 2.6.3. Intracellular Disposition of the Vectors

A density of 150,000 cells in 1 mL per well was seeded in Millicell EZ slides (Millipore) and incubated at 37 °C and 5% CO_2_ for 24 h. Then, they were treated with 75 µL of vector equivalent to 0.8 µg of CleanCap^TM^ Cyanine 5 EGFP mRNA (5moU) as nucleic acid. After 4 h, the slides were washed with PBS, fixed with PFA 4% and covered with the mounting fluid DAPI-fluoromount-G™, used to label the nuclei. Then, a Leica DM IL LED Fluo inverted microscope (Leica Microsystems CMS GmbH, Wetzlar, Germany) was used to analyze the slides.

#### 2.6.4. Quantification of IL-10

To measure the levels of IL-10 expressed by the cells aft er the addition of the complexes, an Enzyme-linked Immunosorbent Assay (ELISA) kit was carried out. The secreted and intracellular IL-10 were quantified 48 h and 72 h after the addition of the mRNA- and pDNA-bearing vectors encoding human IL-10, respectively. For the secreted IL-10, the medium of each well was removed and centrifuged at 12,000× *g* for 2 min. For the intracellular IL-10, the cells were washed with 300 μL of PBS twice, and then 400 μL of reporter lysis buffer 1× was added. Finally, the plate was frozen to complete the lysis of cell culture. After thawing, each well was detached by a scrapper and the lysate was centrifuged at 12,000× *g* for 2 min at 4 °C. A total of 100 μL of each sample was added to a 96-well plate that was covered with the corresponding capture antibody; then the assay was performed according to the manufacturer’s instructions.

### 2.7. In Vivo Studies

Five-week-old male BALB/cOlaHsd mice, with a weight ranging between 20 and 25 g (Envigo), were employed for the *in vivo* studies.

The use of the mice (license M20/2018/142) was approved by The Animal Experimentation Ethics Committee of the University of the Basque Country UPV/EHU following the Spanish and European Union (EU) laws. All the procedures were followed in accordance. The animals were accommodated under controlled temperature, humidity, and 12 h day-night cycles, with food and water *ad libitum* access.

The mice were anesthetized with 1–2% isoflurane (IsoFlo, Abbott, Madrid, Spain) in air, at a flow rate of 0.5–1 L/min with the aim of preventing distress during experimental manipulation.

The mice were humanely euthanatized by cervical dislocation, and then their eyes were removed. After the enucleation, the eyes were washed in a physiological saline solution, fixed with 4% PFA during 30 min and washed with PBS for 5 min. Then, the eyes were immersed in 30% sucrose in PBS at 4 °C until the eyes precipitated. Then, half of the volume was removed and substituted with Tissue-Tek^®^ O.C.T.^TM^ and shaken at room temperature for 2 h. Finally, the eyeballs were stored in 100% Tissue-Tek^®^ O.C.T.^TM^ to freeze at 80 °C for future studies.

#### 2.7.1. Topical Administration

The formulations described in Table 1, as well as naked mRNA encoding GFP or human IL-10, were viscosized with 1% PVA (85,000–124,000 M_*w*_), and administered to the mice by eye drop instillation. The administration of the nanosystems was carried out in 2 doses over 3 days. In each dose, 3 instillations of 2.5 µL at 3 min intervals were carried out, administering a final dose of 4.5 µg of nucleic acid per day.

#### 2.7.2. Evaluation of Gene Expression

The mice were sacrificed 24 h or 48 h after the last dose depending on the formulation administered. For GFP expression studies, the mice were sacrificed at 48 h, except in the case of mRNA-HA-SLN_EE_ formulation, where the mice were sacrificed both at 24 h and 48 h. For IL-10 expression studies, the mice were sacrificed at 24 h. Then, the eyeballs were extracted, fixed and histologically evaluated by sections of 14 µm on a cryostat (Cryocut 3000, Leica, Bensheim, Germany).

In order to evaluate the gene expression, two different transfection studies were carried out. In the first one, vectors contained the nucleic acid that encodes GFP, whereas in the second one vectors were bearing the nucleic acid that encodes IL-10. Both transfections were evaluated qualitatively by immunofluorescence. Sections were washed with a PB buffer. The samples were blocked and permeabilized employing a solution of 20% PB, 0.3% Triton X-100, 10% goat serum, and water q.s. 100%. Then, the respective primary antibody, anti-GFP or anti-IL-10, was added and incubated for 24 h at 4 °C. Secondary antibody goat anti-rabbit IgG Alexa Fluor 488 was added in both transfection assays after washing for 30 min protected from light. Finally, after washing and drying the samples, they were mounted with DAPI-Fluoromount-G. Tissue sections were examined by a Zeiss LSM800 confocal microscope (ZEISS microscopy, Oberkochen, Germany). The overlapping of fluorescence emission spectra was avoided by sequential acquisition. Six sections for each cornea were analyzed as representations of the entire tissue.

### 2.8. Data Analysis

IBM SPSS Statistics 26 (IBM) software was used to perform the statistical analysis, and the Saphiro–Wilk test and Levene test were employed for the evaluation of homogeneity and variance, and normal distribution of samples, respectively. Student’s t-test was used to compare means from two independent groups and ANOVA for multiple comparisons, followed by Bonferroni or T3 Dunnet post-hoc, depending on the results of the Levene test of homogeneity of variances. *p* < 0.05 was considered statistically significant. Data are shown as mean ± standard deviation (SD).

## 3. Results

### 3.1. Size and Zeta Potential of SLNs and Vectors

Table 2 shows the average size, polydispersity index (PDI), and ζ-potential of the SLNs. The particle size ranged from 93.3 to 307.8 nm and PDI values were lower than 0.3. The superficial particle charge ranged from +21.1 to +68.5 mV. Significant differences (*p* < 0.001) were observed in terms of particle size and superficial charge among all SLNs. SLN_C_ showed the highest particle size and the lowest superficial charge, whereas SLN_HM_ showed the smallest size and the highest superficial charge. The Particle Size Distribution Intensity Diagram of SLN_HM_ has been included as an example in (Supplementary Material Figure S1).

Table 3 shows the size, PDI, and ζ-potential of the SLN-based vectors containing CleanCap^TM^ EGFP mRNA (5moU) or IL-10 mRNA customized by TriLink BioTechnologies. All formulations were prepared with protamine (P). Moreover, an aqueous solution of polysaccharide, either dextran (DX) or hyaluronic acid (HA), was incorporated into the SLN_EE_ and SLN_HM_ vectors. Since SLN_C_ vectors contain DEAE-dextran in their composition, only HA was used to prepare the mRNA-HA-SLN_C_ vector. The particle size of vectors containing EGFP mRNA ranged from 132.3 to 348.4 nm, PDIs were lower than 0.4, and the surface charge ranged from +9.5 to +43.9 mV. A PDI value less than 0.4 is related to the homogeneity in the size of the particles of the sample and it demonstrates a monodisperse sample population, which is considered acceptable for drug delivery [38]. Significant differences (*p* < 0.001) in terms of size were observed in both mRNA-SLN_HM_ formulations and mRNA-HA-SLN_C_ with respect to the rest of the formulations. Regarding the superficial charge, significant differences (*p* < 0.001) were observed between SLN_C_ vectors (with the lowest superficial charge), as well as mRNA-DX-SLN_EE_ (with the highest superficial charge), with respect to the rest of the formulations. Particle Size Distribution Intensity Diagram of mRNA-HA-SLN_HM_ bearing CleanCap^TM^ EGFP mRNA (5moU) has been included as an example in (Supplementary Material Figure S2).

IL-10 mRNA vectors showed a smaller particle size than those prepared with GFP mRNA, ranging from 116.9 to 283.9 nm, with a PDI lower than 0.3 and a superficial charge from +19.4 to +49.2 mV. Significant differences (*p* < 0.001) were observed between the vectors prepared with different SLNs with respect to the rest of the formulations in terms of particle size. Regarding the superficial charge, significant differences (*p* < 0.001) were observed between mRNA-SLN_C_ and mRNA-HA-SLN_C_ with respect to the others. 

Table 4 shows the size, PDI, and ζ-potential of the SLN-based vectors containing either plasmid pcDNA3-EGFP or plasmid pUNO1-hIL10. Only SLN_HM_ were employed. All formulations were prepared with P, DX or HA, and SLNs. The particle size of the vectors bearing pcDNA3-EGFP plasmid ranged from 94.5 to 204.0 nm, PDIs were lower than 0.3, and the surface charge ranged from +26.3 to +43.9 mV. Significant differences (*p* < 0.001) were noticed with regard to particle size and ζ-potential between formulations. In the case of pUNO1-hIL10 vectors, the particle size ranged from 101.1 to 193.7 nm, PDIs were less than 0.3 except for pDNA-HA-SLN_HM_, which was 0.48. The superficial charge ranged from +41.4 to +44.7 mV. Significant differences (*p* < 0.001) were reported in terms of size between formulations.

No significant differences in particle size, PDI, or ζ-potential was observed when SLNs were labelled with Nile Red (data not shown).

### 3.2. Agarose Gel Electrophoresis Assay

**Figure 1:**
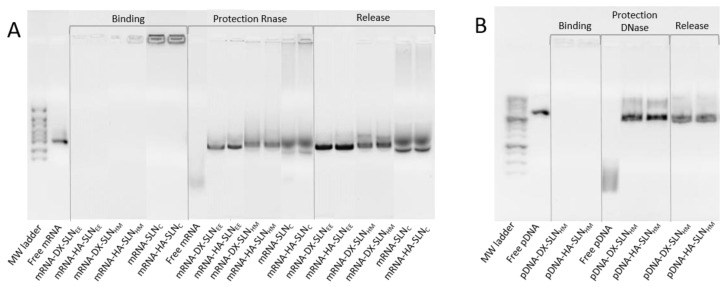
Binding, protection and release capacity of mRNA- and pDNA-based vectors. (**A**) mRNA-SLN_HM_ and mRNA-SLN_C_; (**B**) pDNA-SLN_HM_. DX: dextran; HA: hyaluronic acid; SLN_EE_: solid lipid nanoparticle prepared by emulsification-evaporation method. SLN_HM_: solid lipid nanoparticle prepared by hot-melt emulsification method. SLN_C_: solid lipid nanoparticle prepared by coacervation method.

Figure 1 shows the ability of SLN_HM_ and SLN_C_ vectors to bind, protect, and release mRNA (Figure 1A) and the capacity of SLN_HM_ vectors to bind, protect, and release pDNA (Figure 1B).

Regarding the binding capacity, the absence of bands in both gels and the presence of mRNA (Figure 1A) and pDNA (Figure 1B) on the loading wells indicate that nucleic acid was completely bound to the vector and unable to migrate through the gel. 

Differences in the protection capacity were observed according to the formulation. Bands in the lanes corresponding to vectors prepared with SLN_EE_ and SLN_HM_ were more intense and less faded than those prepared with SLN_C_; these data indicate a higher protection degree of the nucleic acid. Moreover, the presence of the two bands in the SLN_C_ lanes indicates a lower capacity of protection.

In the case of pDNA vectors, both formulations were capable of protecting the nucleic acid against DNase I digestion. All formulations were able to release pDNA after the treatment with SDS.

### 3.3. pH Measurement

Table 5 shows the pH values of mRNA- and pDNA-based vectors formulated as eye drops (1% PVA in HBS (Hanks’ Balanced Salt solution) pH = 7.4). The pH values ranged from 7.13 to 7.44 and did not show significant differences depending on the formulations.

### 3.4. Cell Culture Studies

#### 3.4.1. Transfection Efficacy and Cell Viability

**Figure 2:**
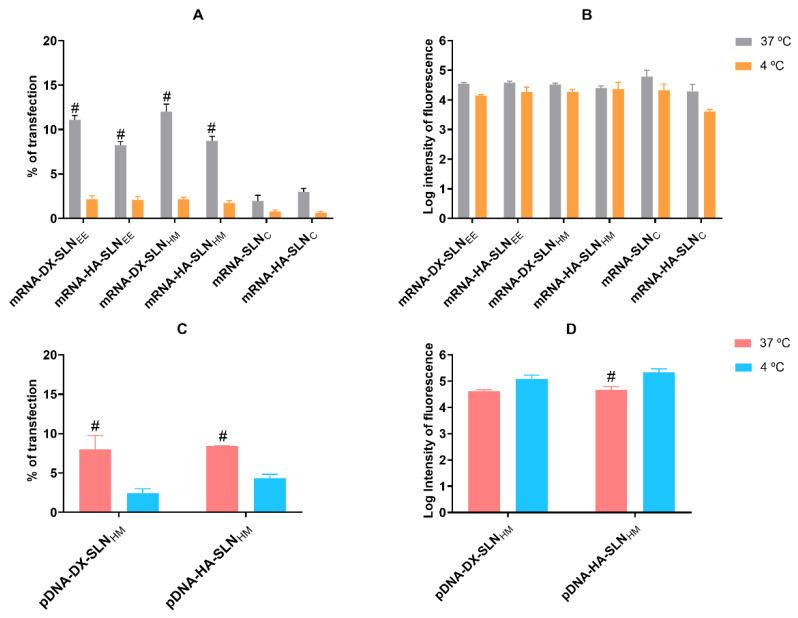
Flow cytometry analysis of transfection efficacy and intensity of fluorescence of HCE-2 cells after the addition of SLN_EE_, SLN_HM_ and SLN_C_ vectors at 37 °C and 4 °C. Percentage of transfection values correspond to the positive fluorescent GFP cells over the total cells. Log of intensity of fluorescence indicates the average intensity of fluorescence per labeled cell. Data are expressed as mean ± standard deviation; *n* = 3. (**A**) Percentage of transfected HCE-2 cells at 37 °C and 4 °C 48 h after administration of mRNA-based vectors. (**B**) Log of intensity of fluorescence of transfected HCE-2 cells 48 h after administration of mRNA-based vectors. (**C**) Percentage of transfected HCE-2 cells at 37 °C and 4 °C 72 h after administration of pDNA-based vectors. (**D**) Log of intensity of fluorescence of transfected HCE-2 cells 72 h after administration of pDNA-based vectors. # *p* < 0.05 with respect to the same vector at 4 °C. DX: dextran; HA: hyaluronic acid; SLN_EE_: solid lipid nanoparticle prepared by emulsification-evaporation method. SLN_HM_: solid lipid nanoparticle prepared by hot-melt emulsification method. SLN_C_: solid lipid nanoparticle prepared by coacervation method.

Figure 2 shows the percentage of transfection and intensity of fluorescence of HCE-2 cells treated with the vectors containing mRNA GFP or pDNA at 37 °C and 4 °C.

Amongst the mRNA-based formulations at 37 °C, the transfection percentage was higher with the vectors prepared with SLN_EE_ and SLN_HM_ (Figure 2A) than with the vectors prepared with SLN_C_. No significant differences were found between nanocarriers prepared with SLN_EE_ and SLN_HM_. With respect to the influence of temperature, the percentage of transfection decreased significantly at 4 °C in all cases. Conversely, the fluorescent intensity (Figure 2B) was similar regardless of the vectors employed and the operating temperature. 

In the case of pDNA-based vectors, the percentage of transfected cells was similar with the two formulations, approximately 8%. At 4 °C, the transfection efficacy was lower, although the intensity of the fluorescence of pDNA-HA-SLN_HM_ increased significantly.

Cell viability at 37 °C was approximately 98% for SLN_EE_ and SLN_HM_, and 90% for SLN_C_. At 4 °C, cell viability was approximately 98% for all formulations.

#### 3.4.2. Cellular Uptake

**Figure 3:**
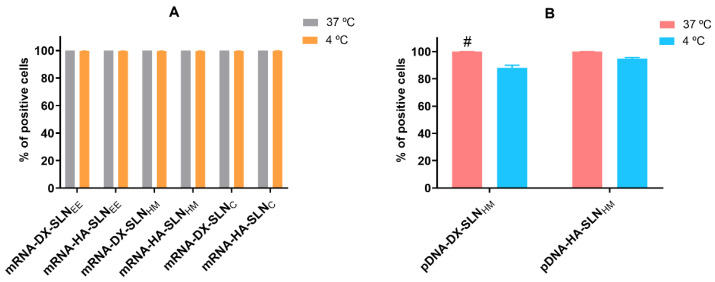
Cellular uptake of vectors using Nile-Red labelled SLNs in HCE-2 cells at 37 °C and 4 °C analyzed by flow cytometry. Percentage of positive cells correspond to the cells which have uptaken the vectors labeled with Nile Red over the total cells. Data are expressed as mean ± standard deviation; *n* = 3. (**A**) Percentage of Nile Red positive HCE-2 cells 2 h after the addition of mRNA-based vectors. (**B**) Percentage of Nile Red positive HCE-2 cells 2 h after the addition of pDNA-based vectors. # *p* < 0.05 with respect to the same vector at 4 °C. DX: dextran; HA: hyaluronic acid; SLN_EE_: solid lipid nanoparticle prepared by emulsification-evaporation method. SLN_HM_: solid lipid nanoparticle prepared by hot-melt emulsification method. SLN_C_: solid lipid nanoparticle prepared by coacervation method.

Figure 3 shows the efficacy of cellular uptake in HCE-2 cells at 37 °C and 4 °C after the addition of mRNA- and pDNA-based vectors labelled with Nile Red. Cell internalization was measured 2 h after the addition of the vectors to the cells cultures.

In the case of mRNA-based vectors, the percentage of positive cells (Figure 3A) was over 99% at both temperatures. In the case of pDNA vectors, the percentage of positive cells was over 99% at 37 °C. In contrast, the percentage of positive cells at 4 °C decreased to 88% for pDNA-DX-SLN_HM_. 

#### 3.4.3. Intracellular Disposition of the Vectors

**Figure 4:**
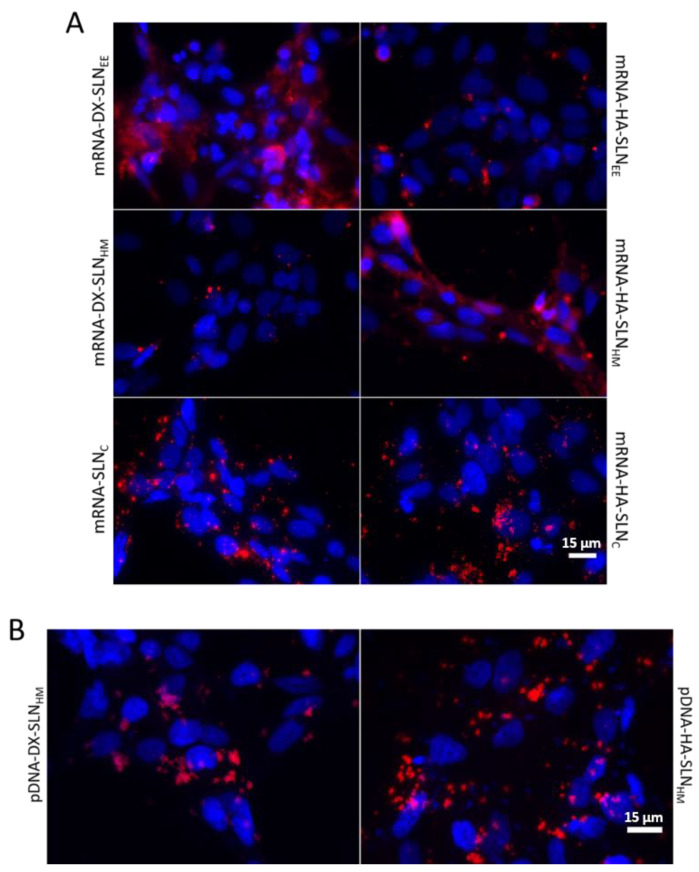
Confocal microscopy analysis of intracellular disposition of mRNA- and pDNA-based vectors 4 h after their addition in HCE-2 cells. (**A**) CleanCap™ Cyanine 5 EGFP mRNA (5moU) vectors formulated with P, DX and HA. (**B**) pcDNA3-EGFP plasmid labeled with Label IT^®^ Cy^®^5 vectors. Blue: nuclei labeled with DAPI. Red: fluorescence signal of nucleic acid labeled with Cy^®^5. Magnification 60×. Scale bar: 15 µm. DX: dextran; HA: hyaluronic acid; SLN_EE_: solid lipid nanoparticle prepared by emulsification-evaporation method. SLN_HM_: solid lipid nanoparticle prepared by hot-melt emulsification method. SLN_C_: solid lipid nanoparticle prepared by coacervation method.

Figure 4 shows the intracellular disposition of mRNA and pDNA in HCE-2 cells, represented by the red fluorescence signal. Depending on the formulation, a difference in the disposition of nucleic acid was observed. 

The mRNA appeared dispersed along the cytoplasm when formulated in DX-SLN_EE_ and HA-SLN_H_ (Figure 4A). However, in the case of the remaining vectors, it appears as dots, indicating that it is more condensed.

The pDNA in the cells treated with the vectors appeared highly condensed and near the nucleus (Figure 4B); no difference in the disposition of the pDNA was observed between the two nanocarriers.

#### 3.4.4. Quantification of IL-10

**Figure 5:**
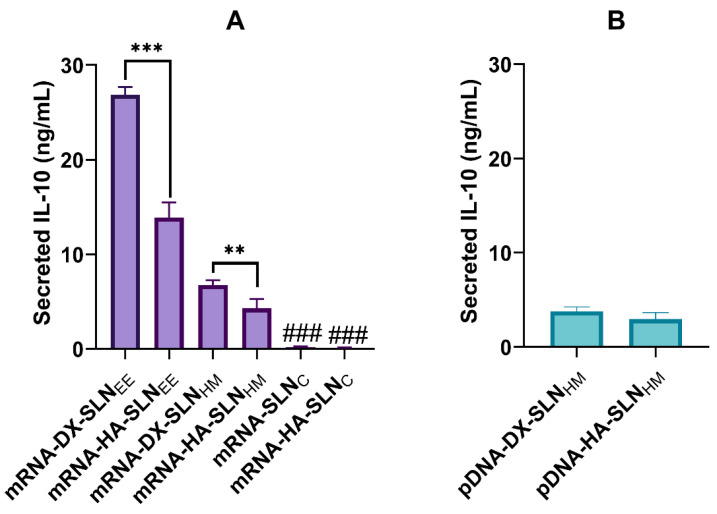
Levels of secreted IL-10 by HCE-2 cells after the administration of SLN-based vectors bearing IL-10 mRNA and pUNO1-hIL10 plasmid. (**A**) Concentration of secreted IL-10 48 h after the administration of mRNA-based vectors. (**B**) Concentration of secreted IL-10 72 h after the administration of pDNA-based vectors. ### *p* < 0.001 with respect to the mRNA-SLN_EE_ and mRNA-SLN_C_ formulations. ** *p* < 0.01 with respect to the other formulation. *** *p* < 0.001 with respect to the other formulation. DX: dextran; HA: hyaluronic acid; SLN_EE_: solid lipid nanoparticle prepared by emulsification-evaporation method. SLN_HM_: solid lipid nanoparticle prepared by hot-melt emulsification method. SLN_C_: solid lipid nanoparticle prepared by coacervation method.

Figure 5 shows the levels of secreted and intracellular IL-10 in HCE-2 cells 48 h and 72 h after the addition of mRNA- and pDNA-based vectors, respectively. The mRNA-vectors induced greater secretion of IL-10 than did the pDNA-based vectors. The basal production of non-treated cells was not detectable. In the case of the mRNA-based vectors, SLN_EE_ formulations were the most effective, while SLN_C_ vectors showed the lowest secreted IL-10 levels.

Intracellular levels of the cytokine were close to 10 pg/mL with all formulations except pDNA-HA-SLN_HM_, reaching 30 pg/mL (Supplementary Material Figure S3). 

### 3.5. In Vivo Studies

#### 3.5.1. *In Vivo* Transfection with mRNA and pDNA Encoding GFP

**Figure 6:**
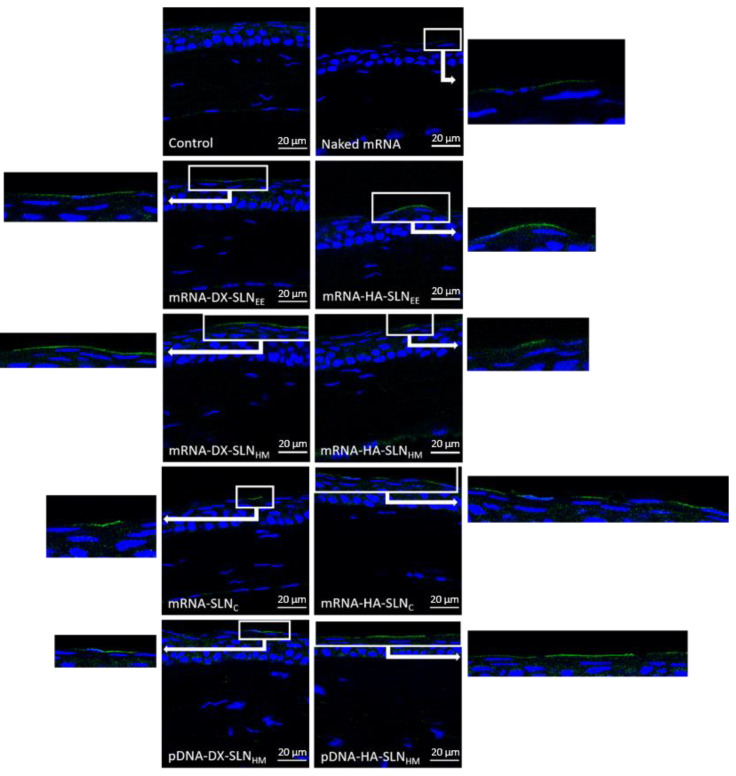
*In vivo* corneal transfection in mice 48 h after the administration of mRNA- and pDNA-vectors encoding GFP with the viscosifier PVA (63×). Blue: nuclei stained with DAPI. Green: GFP detected by immunofluorescence with the secondary antibody labeled with Alexa Fluor 488. Scale bar: 20 µm. DX: dextran; HA: hyaluronic acid; SLN_EE_: solid lipid nanoparticle prepared by emulsification-evaporation method. SLN_HM_: solid lipid nanoparticle prepared by hot-melt emulsification method. SLN_C_: solid lipid nanoparticle prepared by coacervation method.

Vectors bearing CleanCap^TM^ EGFP mRNA (5moU) or plasmid pcDNA3-EGFP combined with PVA were topically administered as eye drops to mice in order to assess the capacity of transfection in the corneal epithelium. Figure 6 shows representative images of the corneas 48 h after being transfected with the different vectors. A 48 h time interval between administration of the formulations and animal sacrifice was initially used, in order to compare the effects of fast acting mRNA and slow acting pDNA vectors.

GFP was detected in 100% of the sections analyzed. All formulations were able to transfect and produce GFP in the corneal epithelium. GFP produced by naked mRNA was difficult to observe, whereas the intensity of fluorescence of GFP was higher when mRNA was formulated in the vectors.

GFP produced by mRNA-DX-SLN_EE_ and mRNA-DX-SLN_HM_ was localized continuously along the epithelium surface. In the case of mRNA-HA-SLN_EE_, mRNA-HA-SLN_HM_, and mRNA-SLN_C_, uninterrupted segments of GFP were observed. In contrast, GFP in the corneas transfected with mRNA-HA-SLN_C_ was localized discontinuously.

Regarding transfection of the corneas with the nanocarriers prepared with pDNA, GFP was detected in a wider area with pDNA-HA-SLN_HM_ than with pDNA-DX- SLN_HM_. pDNA-based formulations were used as controls for the mRNA-based ones; naked pDNA (GFP and IL-10) was previously evaluated [32].

Furthermore, by analyzing the corneas of the mice treated with selected formulations (mRNA-HA-SLN_EE_) after 24 h, it was observed that GFP was detected in a larger surface area and with a higher intensity of fluorescence than after 48 h (Supplementary Material Figure S4). Considering these promising results with mRNA-based vectors, indicating a faster onset of action than initially expected, the corneas treated with the vectors bearing mRNA-IL-10 were evaluated at 24 h post administration. Indeed, a 24 h timeframe, which is required because a quick expression of IL-10 would help to deal with the progression of the inflammatory disease, would be too short for pDNA expression.

#### 3.5.2. *In Vivo* Transfection with mRNA Encoding Human IL-10

**Figure 7:**
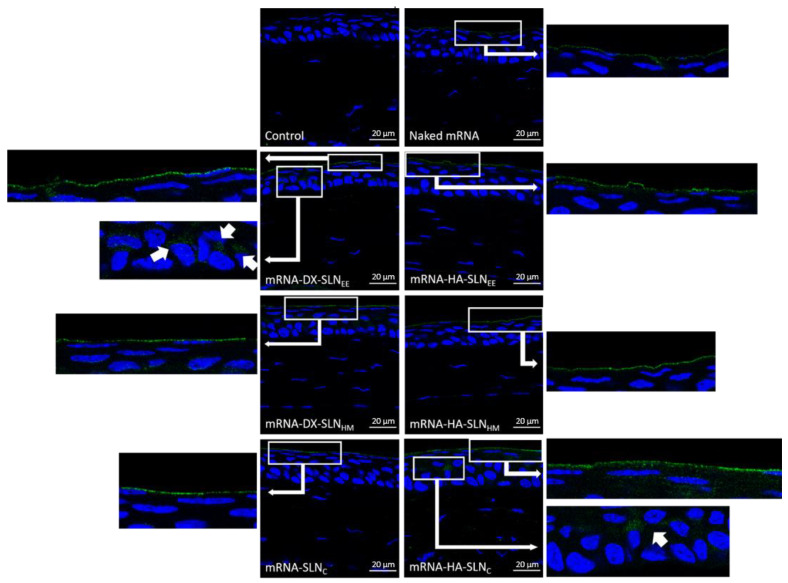
*In vivo* corneal transfection in mice 24 h after the administration of mRNA-vectors encoding human IL-10 with the viscosifier PVA (63×). Blue: nuclei stained with DAPI. Green:IL-10 detected by immunofluorescence with the secondary antibody labeled with Alexa Fluor 488. Scale bar: 20 µm. DX: dextran; HA: hyaluronic acid; SLN_EE_: solid lipid nanoparticle prepared by emulsification-evaporation method. SLN_HM_: solid lipid nanoparticle prepared by hot-melt emulsification method. SLN_C_: solid lipid nanoparticle prepared by coacervation method.

mRNA vectors encoding IL-10 viscosized with PVA were administered to mice as eye drops. The transfection efficacy of vectors was analyzed qualitatively 24 h after the last administration (Figure 7).

IL-10 was observed continuously along the corneal epithelium in all analyzed sections. The intensity of the fluorescence signal was higher when the corneas were treated with the nanosystems than in the case of the naked mRNA. Slight differences were found among the different vectors, although with mRNA-DX-SLN_EE_ and mRNA-HA-SLN_C_, IL-10 was also detected in deeper corneal layers underlying the epithelium (arrows in magnification).

## 4. Discussion

The development of new nanomedicine products for mRNA-based therapies has emerged as an attractive and promising tool in the field of advanced therapies. In this study, we have developed nanovectors based on SLNs for nucleic acid delivery. Three different methods were used to prepare the SLNs: SLN_EE_, prepared by evaporation/emulsification, SLN_HM_ obtained by hot-melt emulsification, and SLN_C_ prepared by coacervation. 

The preparation method influenced the physicochemical features of the SLNs, in terms of particle size and surface charge. In particular, SLN_EE_ and SLN_HM_, made up with the same chemical components but prepared by different methods, varied in particle size. Indeed, it ranged from 90 nm in the case of the hot emulsification procedure, which avoids the use of organic solvents, but involves a high operating temperature, to 200 nm in the case of the evaporation/solvent method. The solvent-free coacervation method, which exploits different compositions compared to the previously mentioned techniques, led to the highest particle size.

The final vectors were made up by electrostatic interactions among SLN, nucleic acid (mRNA or pDNA), and different ligands. Differences regarding the physicochemical characteristics of the SLN may lead to variations in the arrangement of the remaining components, since they are adsorbed on the SLN’s surface to form the final vector. Therefore, the transfection capacity can be influenced. Firstly, the genetic material was condensed with P, which contributes to binding and protecting the nucleic acid extra and intracellularly due to its cationic character [39,40]. Secondly, if required, HA or DX were added; these polysaccharides influence the interactions with the target cells and the intracellular disposition of the nucleic acid [41,42,43]. An additional advantage of the inclusion of one of these polysaccharides is long-term stability for mRNA-SLN-based formulations [33]. 

For corneal transfection, small-sized particles between 10 and 1000 nm reduce eye irritability after topical administrations. Moreover, they show mucoadhesive properties, which help to prolong the residence time and, consequently, to increase the drug bioavailability in the ocular tissues [29,44]. The vectors prepared in our work showed a mean size lower than 300 nm and a positive superficial charge, which facilitates cellular uptake [30] and prolongs the retention time at the corneal epithelium, thanks to the electrostatic interactions with the negatively charged ocular surface [45].

A successful transfection depends upon the balance between the protection provided by the nanosystem to the nucleic acid against degradation, and its capacity to unpack and release the same inside the cell. The preparation method and the composition of the formulations, especially the presence of different ligands, have an important influence on the mRNA condensation and consequently, on the binding, protection, and release from the vector. Electrophoresis on agarose gel (Figure 1) showed that the SLNc vectors presented a weak protection capacity against external agents, and a low release ability. In the case of mRNA-SLN_EE_ and mRNA-SLN_HM_ vectors, differences in the condensation degree were observed (Figure 4). Nevertheless, both formulations protected and released the mRNA effectively (Figure 1). By contrast, no differences in condensation, protection, release capacity, and intracellular disposition of the genetic material were observed in pDNA vectors. Therefore, mRNA seems to be more sensitive to the formulation-related factors than does pDNA.

The interaction between the formulation components and target cells condition the internalization process, and therefore, the intracellular disposition, the endosomal escape and, consequently, the transfection capacity of the systems [41,43,46]. The multi-component nanosystem developed in this work could be adapted to overpass the limiting barriers at the intracellular level, providing an appropriate nucleic acid packaging in the target cells. Endocytosis is the most common mechanism used by SLNs to enter inside the cells. It is an energy- and temperature-dependent process, associated with endosomal/lysosomal pathways of cellular trafficking. Depending on the lipid composition and the physicochemical characteristics of the non-viral vector, the predominant pathway may be different [47,48]. It is well-known that all energy-dependent uptake mechanisms are inhibited by cold temperatures [49]. In the present work, the influence of temperature (37 °C vs. 4 °C) on cellular uptake and transfection efficacy was studied in the HCE-2 cells. Cellular uptake remained stable at both temperatures, which indicates that not only energy-dependent but also energy-independent entry mechanisms are undertaken. Conversely, the percentage of transfected cells decreased significantly at 4 °C, whereas the intensity of fluorescence, indicative of the amount of protein produced by transfected cells, remained almost stable. Therefore, at cold temperatures the few transfected cells are able to produce a higher amount of protein. These results show that the transfection of HCE-2 cells is favoured by energy-dependent mechanisms, although the production of protein seems to be more efficient when the vectors are taken up by energy-independent mechanisms.

The transfection capacity of mRNA- and pDNA-based vectors was similar in terms of transfected cells and intensity of fluorescence (Figure 2). Contrarily to pDNA, mRNA bypasses one of most limiting steps: it does not need to reach the nucleus to transfect, and therefore, it is expected to lead to a faster transfection rate. Noteworthy, our results seem to indicate that the bottleneck for a successful transfection in corneal epithelial cells is before the nuclear entry; as a consequence, the intra-cytoplasmic behaviour of our nanosystems seems to be the limiting step for the transfection.

Transfection studies with the nanovectors bearing pDNA or mRNA encoding the anti-inflammatory cytokine IL-10 were also carried out in HCE-2 cells. The IL-10 was measured in the culture media and at the intracellular level. The mRNA vectors most efficient in terms of IL-10 production were those containing the SLNs prepared by the solvent/evaporation method. For the same kind of SLNs (SLN_EE_ or SLN_HM_), those containing DX were more effective than those containing HA. The mRNA-vectors, including the SLNs prepared by coacervation, hardly produced IL-10. The levels of IL-10 secreted by cells treated with the vectors prepared with SLN_HM_ and either mRNA or pDNA were similar (Figure 5). It is expected that levels over 0.8 ng/mL of IL-10 would exert the anti-inflammatory effect [50]. In previous works, we showed that SLNs-based systems bearing pDNA were able to produce up to 10 ng/mL of IL-10 in HCE-2 cells [8]. In our case, the IL-10 levels obtained with mRNA-SLN_EE_ were higher; in particular, the most effective formulation, mRNA-DX-SLN_EE_, showed IL-10 levels almost three-folds higher. 

The formulation of nanodelivery systems plays a crucial role in the development of medicinal products based on gene therapy, and specifically, in ocular gene therapy. An optimal ophthalmic drug formulation should comply to an adequate bioavailability, an increased permeability, an improved stability against degradation, a prolonged retention on the eye surface, and an augmented interaction with the cornea and targeted delivery [51]. Indeed, due to the pseudoplastic properties of the tear fluid, the inclusion of thickening agents could be advantageous in order to increase the corneal retention time and ocular bioavailability [52]. For the *in vivo* studies, the thickening agent PVA was added to the vectors. The non-ionic and synthetic biodegradable hydrophilic polymer PVA [53] is approved by the FDA for use in ophthalmic formulations [54]. PVA has been widely used because of its muco-mimetic properties, high water retention capacity, oxygen permeability, and low toxicity [55]. These properties confer to our nanosystems the ability to increase the residence time, and consequently improve the ocular bioavailability, reducing the drainage from lachrymal fluid [56,57]. In early studies, our group showed that the combination of SLN-based vectors with PVA provided a higher retention on the cornea [32]. Ophthalmic formulations should have the pH of the lacrimal fluid, or a pH within the range of the ocular comfort range, in order to ensure a good tolerance [58,59]. The ocular pH ranges from 6.6 to 7.8; it is reported that a pH value of an ocular preparation ranged outside 5.0-8.5 causes extra lachrymation and decreases the ocular residence time [60]. Our formulations showed pH values within the ocular tolerance range, from 7.1 to 7.5 (Table 5).

*In vivo* studies in mice were first carried out to evaluate the formulations containing mRNA or pDNA for GFP expression. Since GFP, once produced, remains at the intracellular level, it allowed us to identify the corneal layers where transfection occurs, after the instillation on the mice ocular surface with these vectors. Since the cornea is a complex structure, mRNA-delivery systems are engineered to induce the therapeutic protein expression in the cornea, specifically in the stratified and renewable epithelial layer, where a high number of cells can be transfected. Another alternative could be the transfection of the innermost layer of the cornea, the endothelial layer, associated with a difficult accessibility. Indeed, this layer contains a low number of cells that do not undergo division and gene expression could be maintained for longer times. To this aim, DNA could be most advantageous, since it provides a more persistent transgene production than mRNA. However, although we have previously shown the capacity of DNA-based formulations to transfect the cornea [8], mRNA possesses several advantages that could make it a better option for corneal inflammation management. Indeed, mRNA shows a better safety profile than DNA; the encoded protein is earlier produced, and its expression is transitory, which makes the behaviour of this molecule easier to predict. Moreover, taking into account that the corneal epithelium only needs 7 to 14 days to achieve a complete renewal [61], a short-term expression of the protein is required. 

All mRNA-based formulations included in this experimental work transfected the cornea *in vivo* and showed a higher intensity of fluorescence than naked mRNA. Thus, the SLNs resulted necessary to obtain a high transfection efficiency. These formulations were able to transfect only the epithelial cells but not the inner layers of the cornea, regardless of their different particle size. The intensity of fluorescence of GFP observed in the HCE-2 cells *in vitro*, representative of the protein production, was similar for all formulations, and it seems to correlate better with the *in vivo* results than with the percentage of transfected cells *in vitro*. Nevertheless, the lack of a strict correlation between *in vitro* and *in vivo* studies [62,63,64,65] highlights the necessity to perform the latter ones at the earliest phases of the pharmaceutical development process, in order to perform adequate selection and optimization of candidate formulations.

Finally, we evaluated the capacity of our formulations to induce the production of the anti-inflammatory cytokine IL-10 [51]. In view of the fact that IL-10 is a secreted protein, it may be produced in the epithelial corneal cells and diffuse through the cornea to reach deeper layers. Moreover, for corneal inflammation management, a quick expression of IL-10 would help to deal with the progression of the disease. In this context, we administered the formulations for 3 days and, 24 h after the last administration, the presence of IL-10 in the cornea was assessed. When mice were treated with an mRNA-DX-SLN_EE_ vector, the best performing formulation in *in vitro* experiments in HCE-2 cells (Figure 5), the interleukin was even observed in the deeper layers of the epithelium (Figure 7). mRNA-HA-SLN_C_ vectors also showed a high capacity to produce IL-10, despite the low efficacy observed *in vitro*. It should be considered that the transfection efficacy of the nanovectors could be increased in inflamed corneas, as the histological structure of corneal layers would be altered and disorganized, resulting in a potentially increased permeation of the nanosystems into the tissue. Within this concern, in previous studies with SLN-based nanosystem containing pDNA as the nucleic acid, vectors prepared with the polysaccharide HA showed the highest transfection efficacy [32]. pDNA and mRNA differ not only in their physico-chemical characteristics, but also in terms of their intracellular barriers to overcome in order to achieve protein production. Our results confirm the necessity of the adaptation of the nanovector to the target cells and to the nature of the nucleic acid, in order to obtain an effective transfection.

## 5. Conclusions

The nature of the nucleic acid and the target cell are key points for the design and optimization of nanosystems aimed at ocular gene delivery, with mRNA being more sensitive to the formulation-related factors than pDNA. Nanomedicinal products design is based, initially, on *in vitro* studies. We have demonstrated that the HCE-2 cells’ transfection is favoured by energy-dependent mechanisms, but the production of protein is more efficient when the vectors are taken up by energy-independent mechanisms. However, the lack of correlation observed between *in vitro* and *in vivo* assays has highlighted the necessity to perform *in vivo* studies at the earliest phases of the pharmaceutical development of nucleic acid delivery systems. In the present work, SLNs allowed the ability to obtain a high transfection efficiency *in vivo*. Therefore, topical administration to mice of eye drops containing mRNA formulated in SLNs has shown to be a feasible strategy to tackle corneal inflammation by *de novo* fast IL-10 production from corneal epithelial cells.

## Figures and Tables

**Table 1:** Weight ratios of the formulations.

Name of the Vector	Weight Ratio
mRNA-DX-SLN_EE_	DX:P:mRNA:SLN_EE_ 1:0.25:1:5
mRNA-HA-SLN_EE_	HA:P:mRNA:SLN_EE_ 0.5:0.5:1:5
mRNA-DX-SLN_HM_	DX:P:mRNA:SLN_HM_ 1:0.25:1:5
mRNA-HA-SLN_HM_	HA:P:mRNA:SLN_HM_ 0.5:0.5:1:5
mRNA-SLN_C_	P:mRNA:SLN_C_ 2:1:10
mRNA-HA-SLN_C_	HA:P:mRNA:SLN_C_ 0.5:2:1:10
pDNA-DX-SLN_HM_	DX:P:pDNA:SLN_HM_ 1:2:1:5
pDNA-HA-SLN_HM_	HA:P:pDNA:SLN_HM_ 0.5:2:1:5

SLN: Solid Lipid Nanoparticle. DX: dextran. HA: hyaluronic acid. P: protamine.

**Table 2:** Physical characterization of solid lipid nanoparticles (SLNs).

SLNs	Size (nm)	PDI	ζ-Potential (mV)
SLN_EE_	198.7 ± 2.0	0.26 ± 0.01	+57.8 ± 1.7
SLN_HM_	93.3 ± 0.4	0.28 ± 0.01	+68.5 ± 0.7
SLN_C_	307.8 ± 3.5	0.17 ± 0.01	+21.1 ± 0.8

DX: dextran; HA: hyaluronic acid; SLN_EE_: solid lipid nanoparticle prepared by emulsification-evaporation method. SLN_HM_: solid lipid nanoparticle prepared by hot-melt emulsification method. SLN_C_: solid lipid nanoparticle prepared by coacervation method. PDI: polydispersity index. Data are expressed as mean ± standard deviation; *n* = 3.

**Table 3:** Physical characterization of mRNA-based vectors.

	Size (nm)	PDI	ζ-Potential (mV)
**CleanCap^TM^ EGFP mRNA (5moU)**			
mRNA-DX-SLN_EE_	241.7 ± 4.8	0.29 ± 0.01	+43.9 ± 0.25
mRNA-HA-SLN_EE_	287.2 ± 1.8	0.36 ± 0.01	+37.7 ± 0.3
mRNA-DX-SLN_HM_	132.3 ± 1.9	0.25 ± 0.01	+38.0 ± 1.3
mRNA-HA-SLN_HM_	132.4 ± 1.6	0.20 ± 0.01	+32.7 ± 0.3
mRNA-SLN_C_	292.0 ± 1.2	0.17 ± 0.02	+9.5 ± 0.4
mRNA-HA-SLN_C_	348.4 ± 2.9	0.26 ± 0.01	+13.2 ± 0.6
**IL-10 mRNA**			
mRNA-DX-SLN_EE_	180.1 ± 1.1	0.21 ± 0.00	+49.2 ± 0.3
mRNA-HA-SLN_EE_	199.4 ± 0.7	0.21 ± 0.00	+46.3 ± 0.1
mRNA-DX-SLN_HM_	116.9 ± 0.9	0.25 ± 0.00	+42.1 ± 2.0
mRNA-HA-SLN_HM_	121.3 ± 1.0	0.24 ± 0.00	+41.7 ± 0.2
mRNA-SLN_C_	242.8 ± 1.7	0.25 ± 0.01	+19.4 ± 0.9
mRNA-HA-SLN_C_	283.9 ± 4.6	0.26 ± 0.02	+19.5 ± 1.6

DX: dextran; HA: hyaluronic acid; SLN_EE_: solid lipid nanoparticle prepared by emulsification-evaporation method. SLN_HM_: solid lipid nanoparticle prepared by hot-melt emulsification method. SLN_C_: solid lipid nanoparticle prepared by coacervation method. PDI: polydispersity index. Data are expressed as mean ± standard deviation; *n* = 3.

**Table 4:** Physical characterization of pDNA-based vectors.

	Size (nm)	PDI	ζ-Potential (mV)
**Plasmid pcDNA3-EGFP**			
pDNA-DX-SLN_HM_	94.5 ± 1.0	0.27 ± 0.00	+43.9 ± 1.01
pDNA-HA-SLN_HM_	204.0 ± 2.8	0.25 ± 0.01	+26.3 ± 0.1
**Plasmid pUNO1-hIL10**			
pDNA-DX-SLN_HM_	101.1 ± 1.1	0.27 ± 0.00	+44.7 ± 0.6
pDNA-HA-SLN_HM_	193.7 ± 15.1	0.48 ± 0.01	+41.4 ± 0.2

DX: dextran; HA: hyaluronic acid; SLN_EE_: solid lipid nanoparticle prepared by emulsification-evaporation method. SLN_HM_: solid lipid nanoparticle prepared by hot-melt emulsification method. SLN_C_: solid lipid nanoparticle prepared by coacervation method. PDI: polydispersity index. Data are expressed as mean ± standard deviation; *n* = 3.

**Table 5:** pH measurements of mRNA- and pDNA-based vectors.

Sample	pH
mRNA-DX-SLN_EE_	7.31 ± 0.04
mRNA-HA-SLN_EE_	7.20 ± 0.16
mRNA-DX-SLN_HM_	7.31 ± 0.04
mRNA-HA-SLN_HM_	7.44 ± 0.21
mRNA-SLN_C_	7.08 ± 0.29
mRNA-HA-SLN_C_	7.13 ± 0.11
pDNA-DX-SLN_HM_	7.39 ± 0.06
pDNA-HA-SLN_HM_	7.53 ± 0.02

## Data Availability

The data presented in this study are available on request from the corresponding author.

## Supplementary Materials

The following are available online at https://www.mdpi.com/article/10.3390/pharmaceutics13091472/s1, Figure S1: Particle Size Distribution Intensity Diagram of SLN_HM_. Figure S2: Particle Size Distribution Intensity Diagram of mRNA-HA-SLN_HM_ bearing CleanCap^TM^ EGFP mRNA (5moU). Figure S3: Intracellular levels of IL-10 in HCE-2 cells after the administration of SLN-based vectors bearing IL-10 mRNA and pUNO1-hIL10 plasmid. Figure S4: *In vivo* corneal transfection in wild type mice 24 h and 48 h after the administration of mRNA-HA-SLN_EE_ with PVA (63×).
