# Supplementary material for: mRNA-Based Nanomedicinal Products to Address Corneal Inflammation by Interleukin-10 Supplementation
Source: Pharmaceutics. 2021 Sep 15;13(9):1472. doi: 10.3390/pharmaceutics13091472 (PMC8466377; doi:10.3390/pharmaceutics13091472)
Supplement: Supplementary file 1 [file pharmaceutics-13-01472-s001.zip › Supplementary Material.pdf]

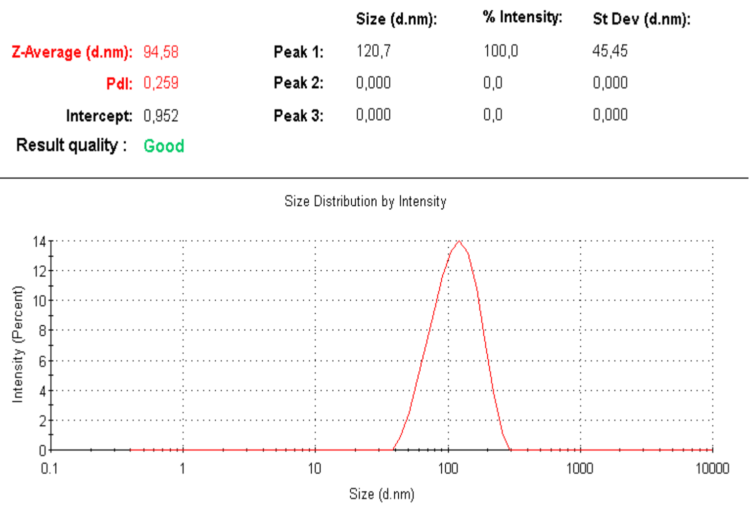

Figure S1. Particle Size Distribution Intensity Diagram of SLN$_{HM}$.

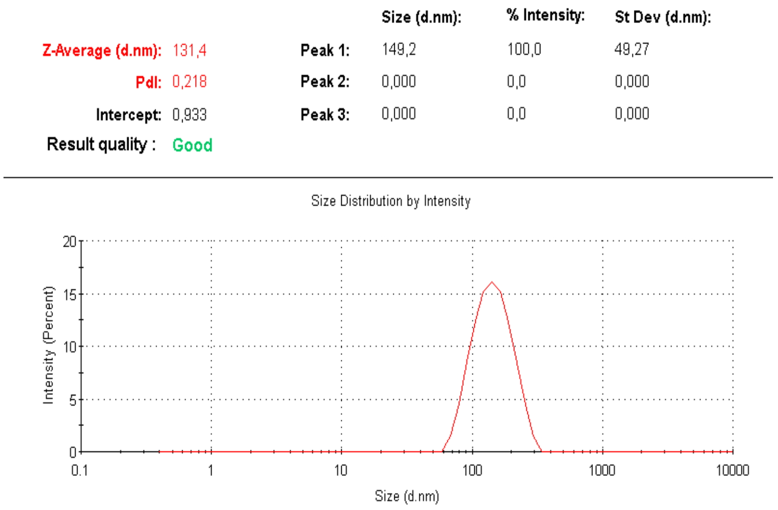

Figure S2. Particle Size Distribution Intensity Diagram of mRNA-HA-SLN$_{HM}$ bearing CleanCap™ EGFP mRNA (5moU).

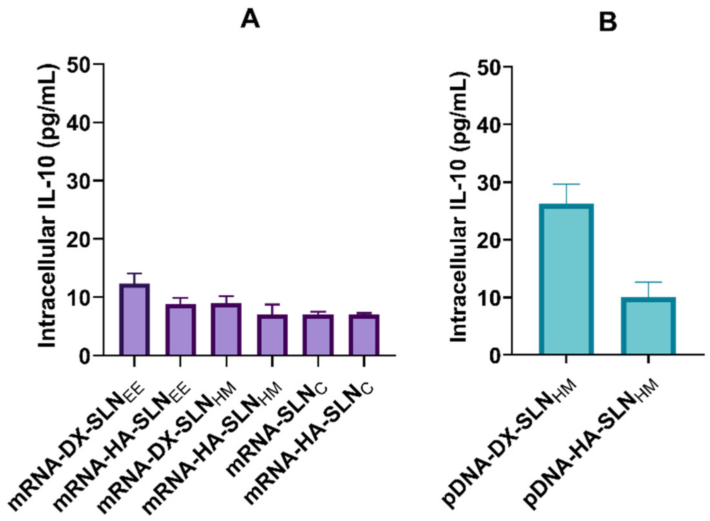

Figure S3. Intracellular levels of IL-10 in HCE-2 cells after the administration of SLN-based vectors bearing IL-10 mRNA and pUNO1-hIL10 plasmid. (**A**) Concentration of intracelullar IL-10 48 h after the administration of mRNA-based vectors. (**B**) Concentration of intracellular IL-10 72 h after the administration of pDNA-based vectors. DX: dextran; HA: hyaluronic acid; SLN_{EE}: solid lipid nanoparticle prepared by emulsification-evaporation method; SLN_{HM}: solid lipid nanoparticle prepared by hot-melt emulsification method; SLN_{C}: solid lipid nanoparticle prepared by coacervation method.

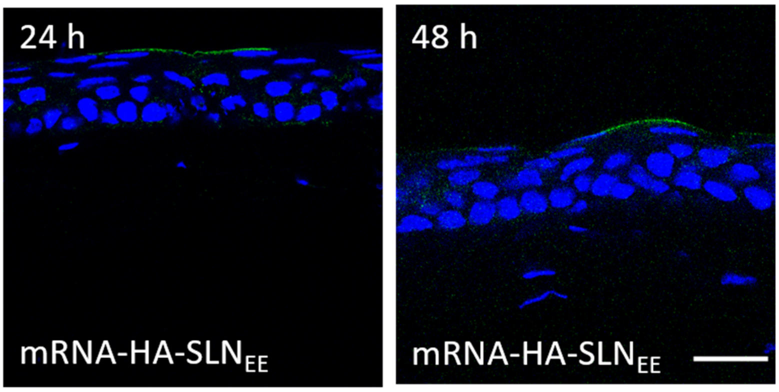

Figure S4. In vivo corneal transfection in wild type mice 24 h and 48 h after the administration of mRNA-HA-SLN_{EE} with PVA (63×). Blue: nuclei stained with DAPI. Green: green fluorescent protein. Scale bar: 20 µm. HA: hyaluronic acid; SLN_{EE}: solid lipid nanoparticle prepared by emulsification-evaporation method.
