# Supplementary figures and images for: mRNA-Based Nanomedicinal Products to Address Corneal Inflammation by Interleukin-10 Supplementation
Source: Pharmaceutics. 2021 Sep 15;13(9):1472. doi: 10.3390/pharmaceutics13091472 (PMC8466377; doi:10.3390/pharmaceutics13091472)

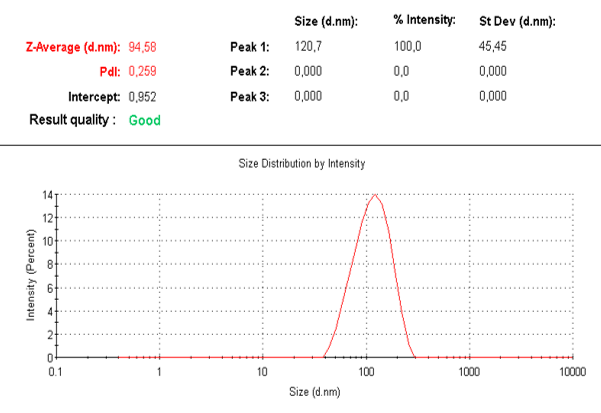

Supplement: Supplementary file 1 [file pharmaceutics-13-01472-s001.zip › Figure S1.tif]

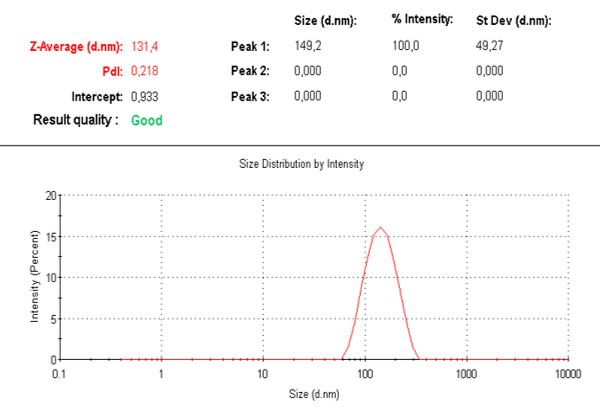

Supplement: Supplementary file 1 [file pharmaceutics-13-01472-s001.zip › Figure S2.tif]

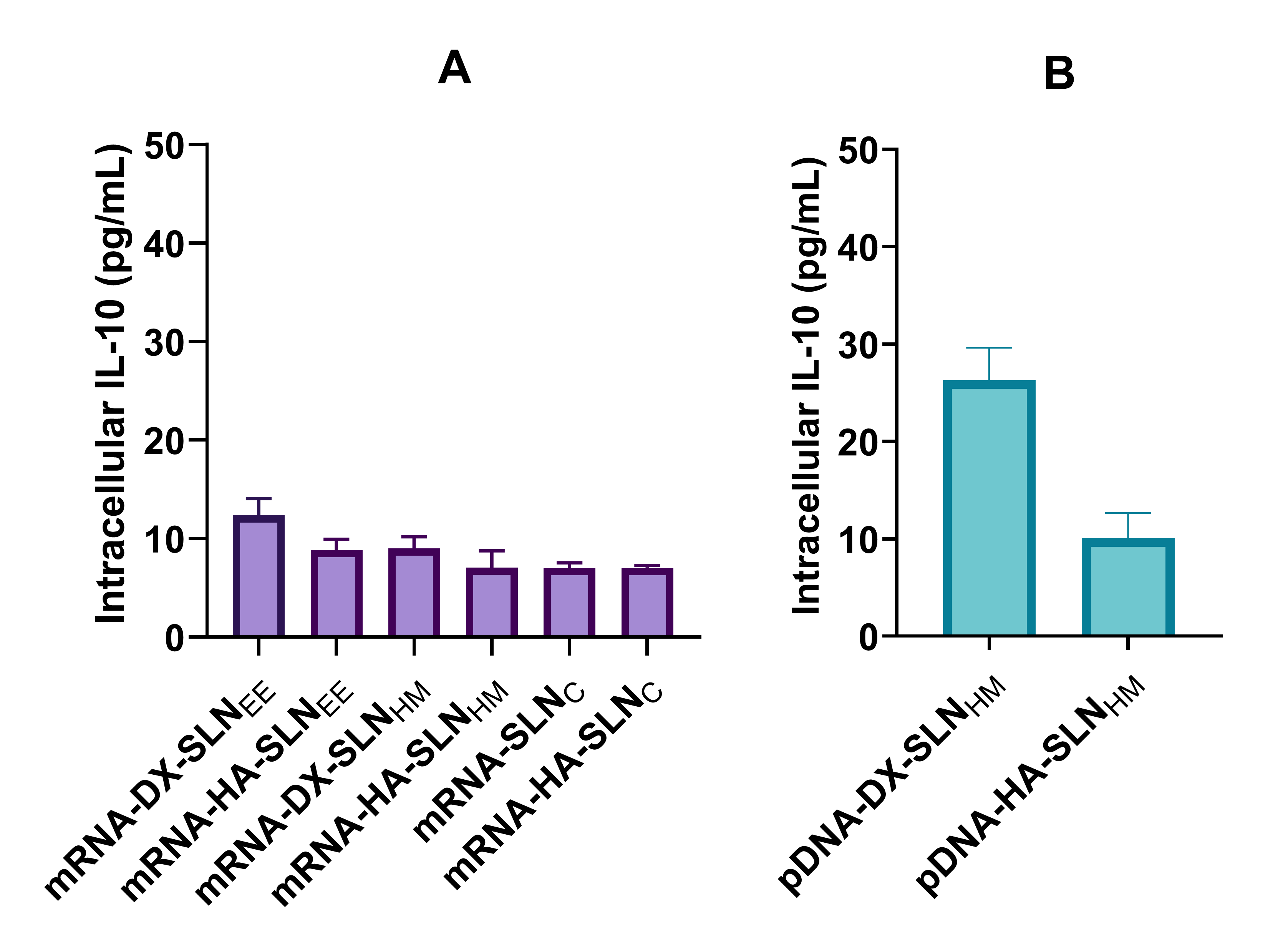

Supplement: Supplementary file 1 [file pharmaceutics-13-01472-s001.zip › Figure S3.tif]

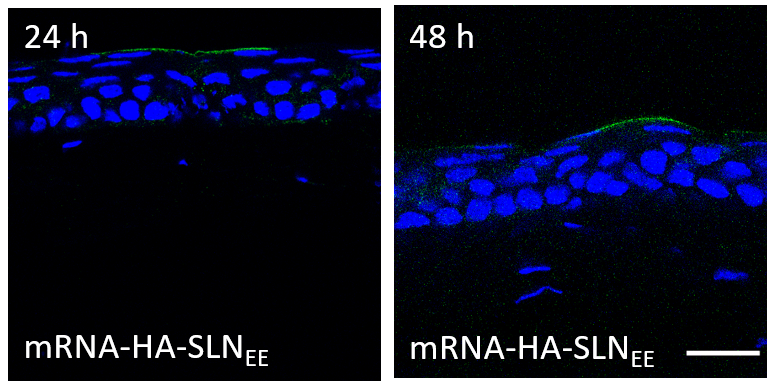

Supplement: Supplementary file 1 [file pharmaceutics-13-01472-s001.zip › Figure S4.tif]
